# Toxicity Assessment and Treatment Options of Diclofenac and Triclosan Dissolved in Water

**DOI:** 10.3390/toxics10080422

**Published:** 2022-07-27

**Authors:** Lenka Wimmerova, Olga Solcova, Marketa Spacilova, Nadija Cehajic, Simona Krejcikova, Petr Marsik

**Affiliations:** 1 Department of Applied Ecology, Faculty of Environmental Sciences, Czech University of Life Sciences Prague, Kamycka 129, 16500 Prague, Czech Republic; cehajic@fzp.czu.cz; 2 Department of Catalysis and Reaction Engineering, Institute of Chemical Process Fundamentals of the CAS, Rozvojova 135, 16502 Prague, Czech Republic; solcova@icpf.cas.cz (O.S.); spacilova.marketa@icpf.cas.cz (M.S.); krejcikova.simona@icpf.cas.cz (S.K.); 3 Department of Food Science, Faculty of Agrobiology, Food and Natural Resources, Czech University of Life Sciences Prague, Kamycka 129, 16500 Prague, Czech Republic; marsik@af.czu.cz

**Keywords:** pharmaceuticals, personal care products, ecotoxicity, *Raphidocelis subcapitata*, photocatalysis, sorption, *Aliivibrio fischeri*, cleaning up

## Abstract

The presence of pharmaceutical and personal care products in water is increasing tremendously nowadays. Typical representatives are diclofenac (DCF) and triclosan (TCS). Acute toxicity of these substances was experimentally assessed using the freshwater algae *Raphidocelis subcapitata* (living, immobilized). The IC_50_ achieved for *R. subcapitata* was 177.7–189.1 mg·L^−1^ for DCF and 5.4–17.2 µg·L^−1^ for TCS, whereas, regarding DCF, the results corresponded to the values observed by other authors. Concerning TCS, the results were lower than predicted and indicated TCSs’ higher toxicity. The immobilized *R. subcapitata* showed comparable results with its living culture for DCF only. Regarding K_2_Cr_2_O_7_ and TCS, the immobilized alga was more sensitive. The DCF and TCF removal from water was tested by sorption, photocatalytic and photolytic processes. TiO_2_ was used as a photocatalyst. Norit and SuperSorbon were used as sorbents based on activated charcoal. The DCF decomposition achieved by both photo-processes was very fast. The starting concentration fell below the detection limit in less than one minute, while bioluminescence on *Aliivibrio fischeri* showed no toxic intermediates formed only in the case of photocatalysis. DCF and TCS removals by sorption were significantly faster on Norit than SuperSorbon, while the bioluminescence inhibition remained insignificant.

## 1. Introduction

Currently, water pollution poses a severe problem, and significant attention has been focused on emerging contaminants. These chemicals include pharmaceuticals, personal care products and various other substances, such as pesticides and endocrine-disrupting compounds [1,2].

They have been found to be ubiquitously present in surface waters, as well as wastewater treatment plant (WWTP) effluents across the world [3,4,5,6]. These substances get into waters mainly at homes and hospitals due to human excretion and/or disposal of unused medication and are released to the aquatic environments in metabolized and/or unmetabolized forms via wastewater discharges [7]. Particularly, effluents from certain small sewage treatment plants were considerably polluted, where concentrations of several pharmaceuticals exceeded 1 µg·L^−1^ [8]. Related to the fact that pharmaceuticals are generally designed to exert an effect on humans, toxic impacts of their residues are considered as or even more serious than those of pesticides.

Some typical representatives of pharmaceuticals are diclofenac (DCF) and triclosan (TCS), antibacterial and antifungal agents used as personal care products additives. Diclofenac belongs to the group of nonsteroidal anti-inflammatory drugs (NSAID) and are one of the most commonly found pharmaceuticals in surface and wastewaters. Its global consumption has been estimated at around 940 tons per year in 2019 [9]. DCF in effluents from WWTP are usually found at levels reaching 1 µg·L^−1^. Several studies have reported chronic toxic effects on fish at these concentrations [10], e.g., liver and kidney damage after 21 days in *Salmo trutta* f. *fario* [11]. Moreover, diclofenac has been found to act in an unspecific manner by nonpolar narcosis and to follow the concept of concentration addition in the growth inhibition test on *Desmodesmus subspicatus*, as well as in the immobilization test on *Daphnia magna* [8]. In many ecotoxicity experiments, performed by standardized methodologies, diclofenac’s effective concentration (EC_50_) or nonobservable/low-observable effective concentrations (NOEC/LOEC) have been established. For example, Ferrari et al. [12] measured EC_50_ 11.45 mg·L^−1^ for *Aliivibrio fischeri* (30 min), 224.3 mg·L^−1^ for *Daphnia magna* and 22.7 mg·L^−1^ for *Ceriodaphnia dubia* (both 48 h). The same authors set the NOEC/LOEC values for *C. dubia* (7 days) 1.0/2.0 mg·L^−1^ and *Danio rerio* (10 days) 4.0/8.0 mg·L^−1^. Similarly, moderate toxicities with EC_50_ in the range from 10 to 100 mg·L^−1^ were observed by other authors [8,13,14]. Basically, *Lemna minor* [8] appeared to be the most sensitive test species for DCF.

Triclosan (TCS), also known as irgasan, is a widely used antibacterial and antifungal agent, which is present as an additive in a variety of consumer products, including soaps, toothpastes, deodorants and cosmetics [15]. This substance is present even in clothes, textile and children’s toys [3]. The release of TCS into aquatic systems causes adverse effects on the environment and biota. Triclosan, being lipophilic, tends to accumulate in animal and human adipose tissue, and has also been detected in urine, blood and breast milk [16]. TCS and its degradation products are persistent, bioaccumulative and toxic, and spread through aquatic and terrestrial food chains [17]. Triclosan has been found in the surface water of various places worldwide in the concentrations ranging from 30 ng∙L^−1^ to 2.3 µg∙L^−1^ [18,19,20] and wastewater effluent from 100 ng∙L^−1^ to 2.7 µg∙L^−1^ [15,17,21]. Moreover, it has been confirmed that TCS tends to concentrate in freshwater sediments in the levels of 0.1 to 53 mg·kg^−1^ dry weight and marine sediments of 0.02–35 μg·kg^−1^ dry weight. Its accumulation in wastewater activated sludge is also considerable, reaching from 0.58 to 15.6 mg·kg^−1^ dry weight [17]. Various authors have established acute toxicity levels of TCS in the range of EC_50_ 0.28 µg·L^−1^ for *Aliivibrio fischeri* (15 min) [22], LC_50_ 260 to 602 µg·L^−1^ for fishes (24–96 h) [16,23], EC_50_ 390 µg·L^−1^ for *Daphnia magna* (48 h) [16] and algae EC_50_ 1.4 to 2.8 µg·L^−1^ for *Desmodesmus subspicatus* (72–96 h) [16,21]. The chronic toxicity data of TCS are still highly limited; however, Orvos et al. [16] and Reiss et al. [21] have measured the NOEC/LOEC values for *D. magna* (21 days) in the levels of 40/200 µg·L^−1^ and for *D. subspicatus* of 0.5/0.69 µg∙L^−1^ (72–96 h) and *Raphidocelis subcapitata* of 0.2/0.4 µg·L^−1^ (72 h) [24]. Algae *R. subcapitata* [25] appeared to be the most sensitive test species for TCS.

Regarding treatment processes, the tertiary treatment approaches have often been proposed to decompose the pharmaceutically active compounds in wastewater [7]. The choice of technology depends on a number of objective factors, such as the type of water and its amount, the load of pathogens, the input composition and the concentration of pharmaceutical and personal care products, including all accompanying pollutants. There are plenty of interesting technological practices already being applied, and even those which could be applied, to all types of water in general (chlorination, ozonation, bioremediation, membrane separation, electrochemical methods, advanced oxidation processes, etc.); however, their scale-up is difficult. A number of hybrid combinations of UV/oxidants, e.g., UV/TiO_2_ + UV/H_2_O_2_ + UV/persuphates + UV/Fenton + O_3_, which may significantly reduce EDs, exist; nevertheless, these have, so far, been more interesting in terms of scientific knowledge, especially regarding the economic point of view [26]. The literature abounds in hundreds of sophisticated procedures; unfortunately, they are realistically feasible only in the laboratory scale.

The use of ultraviolet (UV) radiation [27,28], similar to photocatalysis [29], is effective and reduces pollutant concentrations; however, it can also produce a number of toxic by-products [30]. For example, Salgado et al. [31] have identified numerous transformation by-products of DCF formed during UV photolysis (such as hydroxylated species, quinone-imine and phenyl-acetic acids). Furthermore, the environmental metabolite of TCS methyl-triclosan (MTCS) increases hydrophobicity and also shows a higher bioaccumulation potential [32]. Thus, it is important to design more effective treatment strategies for pharmaceutically active compounds not only to minimize their general discharge to the environment, but also to prevent the discharge of those by-products which are even more harmful than their parent compounds [7]. The sorption process seems to be a suitable candidate even for the scale-up and real application as a tertiary step in water treatment plants. Various types of sorbents have been successfully tested, including specially modified organoclays with cationic surfactants [33], lignite activated cokes [34], and biological activated carbon [35].

In this study, acute toxicity of various DCF and TCS concentrations was assessed by the freshwater microalga *Raphidocelis subcapitata* applied as a living (free) culture and/or in an alginate (immobilized) form. Simultaneously, the removal of DCF and TCS from water by sorption and photo-processes was performed. The rate and efficiency of sorption were compared with contaminants’ disintegration by photocatalytic and photolytic processes, which are considered to be highly effective methods. The article is focused not only on each process efficiency, but also the assessment of a potential risk of treated water due to its toxicity increase. During these experiments, toxicity was continuously monitored on the luminescence bacteria of *Aliivibrio fisheri*.

## 2. Experimental

### 2.1. Materials and Reagents

Diclofenac sodium salt (an analytical standard, C_14_H_10_Cl_2_NNaO_2_, CAS: 15307-79-6); triclosan (a certified reference material, C_12_H_7_Cl_3_O_2_, CAS: 3380-34-5); cyclohexane (for HPLC ≥ 99.9%, C_6_H_12_, CAS: 110-82-7); potassium dichromate (≥97.0%, K_2_Cr_2_O_7_, CAS: 7778-50-9), titanium (IV) isopropoxide (≥97.0%, Ti[OCH(CH_3_)_2_]_4_, CAS: 546-68-9) and Triton X-114 (a laboratory grade, C_27_H_48_O_7.5_, CAS: 9036-19-5), supplied by Sigma-Aldrich (Prague, Czech Republic), were used during the toxicity assessment and treatment experiments.

Ammonium chloride (≥99.5%, NH_4_Cl, CAS: 12125-02-9); boric acid (≥99.5%, H_3_BO_3_, CAS: 11113-50-1); calcium chloride, dihydrate (≥99.0%, CaCl_2_·2H_2_O, CAS: 10035-04-8); cobalt chloride, hexahydrate (98%, CoCl_2_·6H_2_O, CAS: 7791-13-1); copper chloride, dihydrate (≥99.0%, CuCl_2_·2H_2_O, CAS: 10125-13-0); disodium EDTA, dihydrate (≥99.0%, Na_2_EDTA.2H_2_O, CAS: 6381-92-6); ferric chloride, hexahydrate (97.0%, FeCl_3_·6H_2_O, CAS: 10025-77-1); magnesium chloride, hexahydrate (99.0%, MgCl_2_·6H_2_O, CAS: 7786-30-3); magnesium sulphate, heptahydrate (≥98.0%,, MgSO_4_·7H_2_O, CAS: 10034-99-8); manganese chloride, tetrahydrate (≥98.0%, MnCl_2_·4H_2_O, CAS: 13446-34-9); potassium dihydrogen phosphate (≥99.0%, KH_2_PO_4_, CAS: 7778-77-0); sodium bicarbonate (≥99.7%, NaHCO_3,_ CAS: 144-55-8); sodium molybdate, dihydrate (≥99.0%, Na_2_MoO_4_·2H_2_O, CAS: 10102-40-6) and zinc chloride (≥98.0%, ZnCl_2_, CAS: 7646-85-7), all supplied by Sigma-Aldrich (Prague, Czech Republic), and demineralized water (with conductivity of less than 10 μS.cm^−1^) produced in the PURELAB flex 1 equipment supplied by ELGA LabWater (Lane End, UK) were used for the alga growth media preparation.

The activated charcoal Norit^®^ SX 2 pure p.a. (POCH SA, Gliwice, Poland) and SuperSorbon I (Donau Carbon, Frankfurt, Germany) were used during sorption tests. The glass beads used as the carrier for catalyst’s layers (diameter 1.5 mm) were supplied by Ginzel (Lodenice u Berouna, Czech Republic).

**Figure 1:**
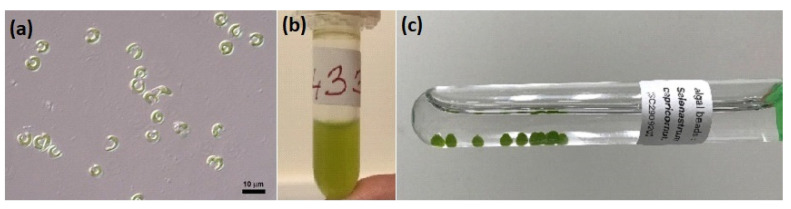
Used forms of *Raphidocelis subcapitata* (formerly *Selenastrum capricornutum*): (**a**) culture SKULBERG 1959/1 (CCALA 433) [36]; (**b**) living (free) inoculum; (**c**) immobilized algal beads.

The freshwater microalga *Raphidocelis subcapitata* (formerly *Selenastrum capricornutum*) was applied in two forms: as a living (free) culture and in an alginate (immobilized) form (Figure 1). The fresh microalga was supplied in a 1.5 mL Eppendorf ampule from the Culture Collection of Autotrophic Organisms (CCALA, Trebon, Czech Republic). The strain no. 433 *Raphidocelis subcapitata* (Korshikov) Nygaard et al. [36] was used. The immobilized alga was supplied commercially as algal beads in 10 mL tubes (MicroBioTests, Gent, Belgium). 

The luminescence bacteria of *Aliivibrio fisheri* (formerly *Vibrio fischeri*) was not purchased independently. The luminescence test was performed in a selected accredited laboratory (see Section 2.2).

### 2.2. Toxicity Assays

The freshwater toxicity assessment on *R. subcapitata* was carried out in accordance with the ISO 8692:2012-ed.3.0 standard on the *Water quality*—*Fresh water algal growth inhibition test with unicellular green algae* and the OECD Guideline—*Test no. 201 Freshwater alga and cyanobacteria*, *growth inhibition test* [37,38]. Both methods are appropriate for chemicals easily dissolvable in water. They are based on the observance of growth of the concentration of algal cells in the test solution and the growth medium through time.

The microalga toxicity assays were performed as 6 tests, each repeated 3 times. Three tests were performed with de-immobilized algal cells and three with the living algal cells. Each was exposed to different concentrations of potassium dichromate (0; 0.3; 0.6; 1.2; 2.4; and 4.8 mg·L^−1^) used as a reference substance, triclosan (0; 1.5; 4.5; 13.5; 40.5; and 121.5 µg·L^−1^) or DCF (0; 50; 100; 200; 400; and 800 mg·L^−1^) separately. Microalga toxicity assays were carried out in 100 mL Erlenmeyer flasks placed on a stainless-steel mixing plate (P-Lab, Prague, Czech Republic) under a fluorescent white light lamp unit (MicroBioTests, Gent, Belgium), with constant illumination of 6000–10,000 lux. The optimal cultivation conditions were maintained within the temperature 23 ± 2 °C by a wall CO_2_/thermo detector (Klimafil, Prague, Czech Republic). The initial concentration of algal cells in each flask was 10,000 c·mL^−1^. In 72 ± 2 h, the concentration of algal cells was measured in each sample with a spectrophotometer instrument Cary 60, at 685 nm wavelength (Agilent, Prague, Czech Republic) 6 times, for better precision.

The average specific growth rate for a period of time was calculated as the logarithmic increase in the biomass through an equation mentioned in the ISO 8692:2012-ed.3.0 standard, as well as the percent inhibition of growth rate. The inhibition concentration (IC_50_) was estimated with the Probit method using the MS Excel program (Microsoft, USA). Regarding the inhibition concentration (IC_50_), the PROAST module calculation (the National Institute of the Netherlands), as a part of the programing language R, was used. The PROAST is a software package used for statistical analyses of dose–response data (‘*PROAST*|*RIVM*’). The achieved data were inserted in the module, and the IC_50_ was calculated by two methods—the exponential method (met1) and the Hill method (met2). The validity of the algal tests was checked by the fulfilment of the following parameters: the average specific growth rate had the minimum value of 1.4 d^−1^, the pH value at the end of the test did not differ more than 1.5 from the starting value of pH, and the calculation of the variation coefficient of the growth rate in the control replicates did not exceed 5%.

The toxicity assay of intermediates (from photocatalysis and photolysis experiments) were determined by the bioluminescence assay on *Aliivibrio fischeri* according to the ISO 11348-2:2007-ed.2.0/Amd.1:2018 standard on the *Water quality*—*Determination of the inhibitory effect of water samples on the light emission of Vibrio fischeri* (*Luminescent bacteria test*)—*Part 2: Method using liquid-dried bacteria* [39]. The luminescence inhibition rate was measured at 15 and 30 min of exposition by the LUMIStox instrument using the Lumistox Luminescent bacteria test (both HACH LANGE, Dusseldorf, Germany). The degree of inhibition was evaluated from the average values of luminescence intensity, which was measured in several replicates by an accredited laboratory of the Povodi Labe Co. (Obristvi, Czech Republic).

### 2.3. Photocatalyst and Sorbent Characterization

**Figure 2:**
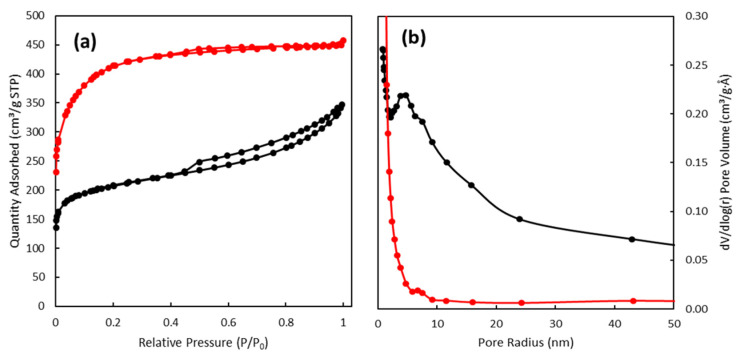
Textural characteristic of used sorbents 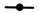 Norit and 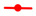 SuperSorbon. (**a**) The isotherm of physical nitrogen adsorption. (**b**) Distribution of mesopores from the adsorption branch of the isotherm of physical nitrogen adsorption.

Textural properties of both sorbents, Norit and SuperSorbon, were determined by nitrogen physisorption using the volumetric instrument ASAP 2020 (Micromeritics, Norcross, GA, USA). The specific surface area (S_BET_) was evaluated by the BET method; the micropore volume (V_micro_) and the mesopore surface area (S_meso_) by the t-plot method with Lecloux–Pirard master isotherm; and pore-size distribution by the advanced BJH method. Nitrogen isotherms of both sorbents revealed a combination of I and IV types of isotherms according to IUPAC classification, with a steep increase at very low *p*/*p*_0_, which indicated the presence of micropores. Both sorbents possessed a high surface area, with a significant portion of micropores together with the clear maxima of the pore radius at 7.6 nm for SuperSorbon and 4.7 nm for Norit. It was evident that textural parameters of SuperSorbon almost doubled those of Norit; however, the particle sizes of applied sorbents, Norit 0.25 mm and SuperSorbon 5 mm, could have significantly influenced the sorption rates, owing to the diffusion of molecules into the binding sites (Figure 2).

**Figure 3:**
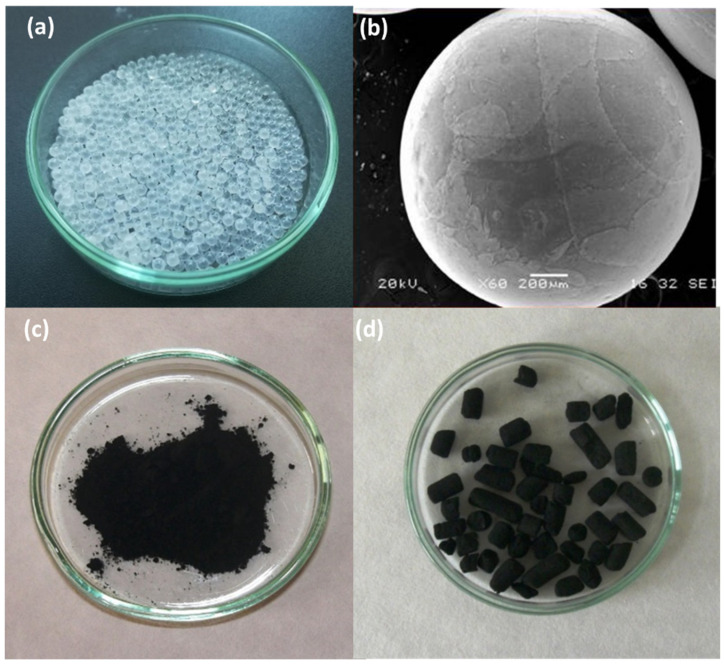
Used photocatalysts and sorbents. (**a**) Glass beads with TiO_2_ layer. (**b**) Glass beads with TiO_2_. (**c**) Activated charcoal Norit. (**d**) Activated charcoal SuperSorbon.

The crystallographic structure of the prepared thin film of TiO_2_ photocatalyst was performed by X-ray diffraction (Panalytical-MRD laboratory diffractometer with the Cu anode). TiO_2_ photocatalyst in the form of four thin layers contained only a pure anatase crystalline phase. The textural properties of TiO_2_ photocatalyst were evaluated for a powder equivalent and are shown in Table 1. Further details can be found at Solcova et al. [40]. The photos of glass beads with TiO_2_ layer together with both sorbents (Norit and SuperSorbon) are shown in Figure 3.

### 2.4. Treatment Experiments

The photolytic and photocatalytic reaction experiments regarding the removal of both pollutants were performed under UV light at normal temperature and under pressure conditions (25 °C; 1 atm). The experiments were accomplished in a batch reactor with the reaction solution volume of 200 mL. The initial concentration of DCF or TCS was 1 mg·L^−1^. The beaker contained a bed of the photocatalyst, glass beads covered by four nanostructured TiO_2_ layers (5 g beads with 2.5 mg active amount of TiO_2_) [40,41]. The UV lamp Philips HOK 4/120 SE, 400 W medium pressure mercury lamp (Philips N.V., Amsterdam, the Netherlands) with a wavelength in the range of 250–420 nm was applied for the photoreactions. It reached several maxima in the whole range, and the intensity of the light measured in the reactor was 62 W·m^−2^ for UV-Vis, 55 W·m^−2^ for UVA, 46 W·m^−2^ for UVB and 108 W·m^−2^ for UVC [40]. To avoid the influence of sorption, the photocatalyst was immersed into the solution and kept in the dark for 15 min. Subsequently, the UV lamp was turned on.

The sorption experiments were realized in a batch reactor using an orbital shaker GFL 3005, 300 rpm (Lauda DR. R. Wobser GmbH & Co. KG, Lauda-Königshofen, Germany) with a particular volume of the reaction solution at 100 mL. The initial concentration of DCF or TCS used was the same as in the photolytic and photocatalytic processes. Activated commercial charcoal Norit and SuperSorbon were chosen to perform sorbent tests. The amount of applied sorbent was 1 g per 100 mL of the solution. The sorption experiments were executed under the normal temperature and pressure conditions (25 °C; 1 atm). Sorbents were separated from the samples by a nylon filter with the pore size of 0.45 μm (Carl Roth GmbH & Co. KG, Karlsruhe, Germany). All reactions were repeated at least three times, and the error never exceeded 5%.

The LC/MS system consisting of a UHPLC chromatographic station Dionex Ultimate 3000 (Thermo Fischer Scientific, Waltham, MA, USA) and a Q-TOF mass spectrometer with ultra-high resolution (>60,000 FSR) and precise molecular weight determination (HRAM) Q-TOF Impact II (Bruker Daltonik, Bremen, Germany) were used for contaminant analyses. The samples were separated using the Acclaim^®^ RSLC 120 C18 column (2.2 μm 120 Å 2.1 × 100 mm, Thermo Fisher Scientific, Waltham, MA, USA) equipped with the Acquity UPLC BEH C18 VanGuard precolumn (Waters, Milford, MA, USA) by gradient elution using 0.1% formic acid mobile phases (A) and methanol (B) at the constant flow rate of 0.3 mL·min^−1^. The gradient started at 30% B; then, it was increased to 100% B in 5 min and held for 3 min, and, finally, was equilibrated to initial conditions by 30% B for 4 min. The column temperature was set at 35 °C.

## 3. Results and Discussion

### 3.1. Toxicity Assay

After 72 ± 2 h of testing, the concentration of algal cells in the samples varied between 20,000 c·mL^−1^ and 2.5 mil. c·mL^−1^, depending on the tested contaminant and its concentration. The determined average growth rate and the average inhibition rate expressed as a percentage can be found in Table 2 for the free algae and Table 3 for the immobilized algae.

**Figure 4:**
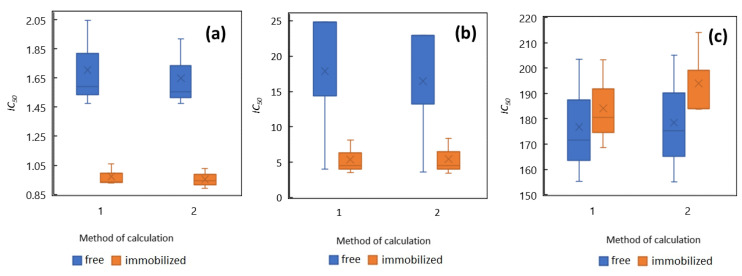
Experimentally measured IC_50_ on *R. subcapitata* (left axis). (**a**) K_2_Cr_2_O_7_ in mg·L^−1^; (**b**) TCS in µg·L^−1^; (**c**) DCF in mg·L^−1^.

The pH measurements before and after testing the control samples, coefficient variation of growth rate and the IC_50_ are displayed in Table 4. The table and Figure 4 reflect the results connected with the two methods used for measuring the IC_50_. The (met1) regards the exponential method (met1) and (met2) the Hill method.

Concerning the table and the graph mentioned above, the average IC_50_ for *R. subcapitata* for DCF 177.7 mg·L^−1^ (free alga) to 189.1 mg·L^−1^ (immobilized alga), and opposite for TCS 5.4 µg·L^−1^ (immobilized alga) to 17.2 µg·L^−1^ (free alga), was achieved. Related to DCF, the results corresponded to the values observed by other authors [13,14], whereas the TCS results from our experiments were lower than expected.

Potassium dichromate (K_2_Cr_2_O_7_) was used as a reference substance during the alga toxicity assay. According to Santos et al. [42], the IC_50_ was established at 0.9 mg·L^−1^ of potassium dichromate. A similar IC_50_ was indicated by our research for the immobilized alga *R. subcapitata*, while the average IC_50_ for the immobilized alga was measured at 1 mg·L^−1^. The growth inhibition related to alga inoculum was significantly lower, 1.7 mg·L^−1^, which means that the fresh living alga were less sensitive to potassium dichromate during our experiments. In the case of diclofenac (DCF), the IC_50_ concentrations were 177.7 mg·L^−1^ for free-living *R. subcapitata*, resp. 189.1 mg·L^−1^ for its immobilized form. These results are fairly similar, unlike the reference dichromate, meaning that the sensitivity of both immobilized and free algae was comparable concerning DCF. However, the experimentally achieved values in this study were higher than the maximum <100 mg·L^−1^ observed by other authors [8,13,14,43]. Triclosan (TCS) was tested in lower concentrations (in µg·L^−1^), since its toxicity is much higher than DCF and the reference substance of potassium dichromate. The experimentally measured IC_50_ was lower for the immobilized algae, with the IC_50_ average of 5.4 µg·L^−1^, contrasting with 17.2 µg·L^−1^ for the living alga. This indicates that, similarly to potassium dichromate, free-living algae are less sensitive to TCS than *R. subcapitata* immobilized in the alginate form. The average IC_50_ of TCS achieved on immobilized alga corresponded to the value of 4.7 µg·L^−1^ established by Tatarazako et al. [44], which was, however, still higher that the values of 0.2/0.4 µg·L^−1^ measured on the *R. subcapitata* by Yang et al. [24].

The previous research, where the sensitivity of immobilized and living algae to heavy metals and pesticides was compared [45,46], showed that immobilized alga was either less sensitive or comparable to living alga. Al-Hasawi et al. [45] explained the lower sensitivity of immobilized algae to heavy metals as an effect of alginate, when alginic acid, alginate, binds strongly to divalent cations, and thus reduces the toxicity of the tested substances. However, this was not confirmed in our study. The immobilized *R. subcapitata* showed only comparable results for DCF, while, for potassium dichromate and TCS, the immobilized alga was controversially more sensitive than its living form. It was assumed that the result was achieved due to the applied precise de-immobilization methodology, within which the released alginate was thoroughly, twice washed out of alga suspension. Thus, there was no reason for alginate properties to be the key factor of the sensitivity of immobilized alga. The differences between immobilized algae beds and living algae cultures were recognized based on the length of lag period. The immobilized algae have a longer lag period. After the lag period was over, the specific growth rate was similar concerning both the immobilized and living algae [47]. This seems logical, since the immobilized algae were chemically treated to be immobilized, and then chemically treated to be de-immobilized, which is uncomfortable for algae. Therefore, they needed more time to adjust to the newly introduced conditions. Moreover, free algae were introduced to the test conditions for the period of three or four days prior to the test; hence, they overcame the lag phase of adjusting. The immobilized algal cells were immobilized in their exponential phase; nevertheless, the de-immobilization procedure and introduction into new conditions could also affect the exponential phase.

### 3.2. Contaminant Removal

Sorption technique was tested as a cheap method. This could solve the issue of water contaminants that are either slowly biodegradable and/or easily biodegradable but their massive consumption leads to continuous emission, which is significantly faster than its environmental removal rate [48]. Simultaneously, the photocatalysis over titanium dioxide, which is known for high efficiency of contaminant removal [49,50,51], and photolysis were performed as comparative methods. Taking into consideration the scaling up and possible future applications, titanium dioxide was used in the form of a thin layer, owing to the fact that TiO_2_ in a powder form is suitable only for laboratory experiments.

**Figure 5:**
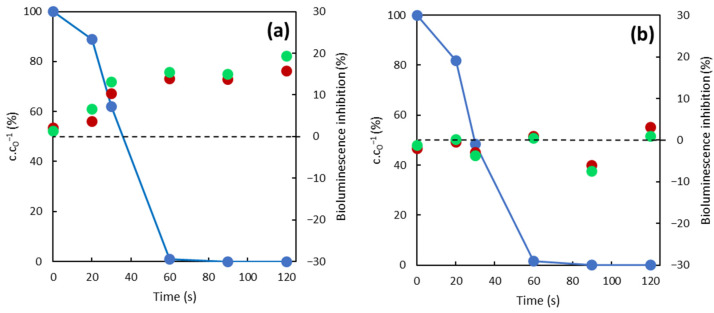
Time dependence of DCF over time (left axis—normalized concentration 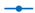 DCF (%); right axis—inhibition of bioluminescence ● after 15 min and ● after 30 min). (**a**) Photolytic reactions; (**b**) photocatalytic reactions.

Normalized diclofenac (DFC) concentration c.c_0_^−1^ (left axis y) together with the bioluminescence inhibition of *A. fischeri* bacteria (right axis y) over time are shown in Figure 5. Evidently, the decomposition of diclofenac was very fast. The initial concentration of diclofenac fell below the detection limit (0.87 ng/mL) in less than one minute for both photo reactions. The bioluminescence inhibition of bacteria represented the toxicity of formed intermediates and was generally low (because of low initial concentration of DCF). The degree of inhibition for photocatalysis remained around zero, which means that no toxic intermediates were formed during the reaction. Contrarily, toxic intermediates were formed in the case of photolysis, and the inhibition of bioluminescence increased over time, which characterizes this method as an environmentally unfriendly process. It corroborates the published results [52,53,54].

**Figure 6:**
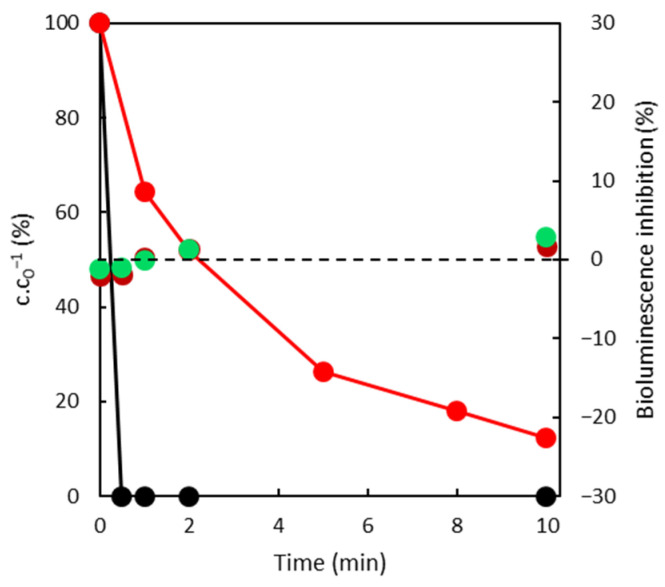
Time dependence of DCF over time on both sorbents 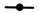 Norit, 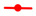 SuperSorbon (left axis—normalized concentration (%); right axis—inhibition of bioluminescence ● after 15 min and ● after 30 min).

Figure 6 reflects DFC removal by sorption on SuperSorbon and Norit. Obviously, the efficiency of DFC removal by sorption on Norit was 20 times faster than on SuperSorbon, and even two times faster regarding the photocatalysis reaction. The differences between applied sorbents relate to the particle sizes (Norit 0.25 mm and SuperSorbon 5 mm). The internal diffusion occurred in the case of SuperSorbon and significantly affected the rate of sorption. The inhibition of bioluminescence during the experiment remained at zero, which was predictable for the sorption process.

**Figure 7:**
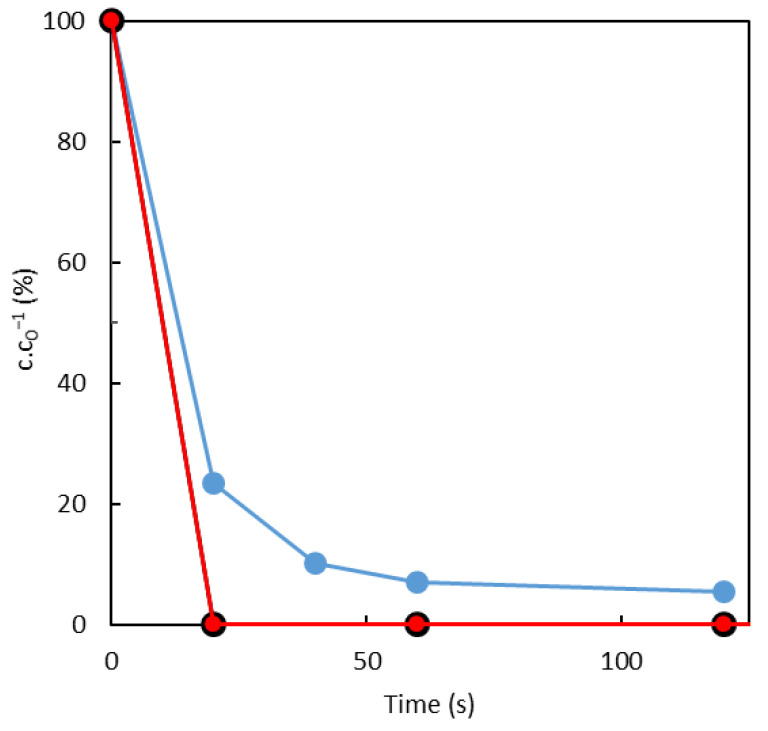
Time dependence of concentration of TCS over time by a sorption on 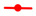 SuperSorbon, 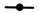 sorption on Norit and 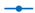 decomposition by photocatalysis.

Concerning triclosan (TCS) removal, the efficiency of both tested systems, sorption and photocatalysis, is shown in Figure 7. It is evident that both processes were really fast. The TCS concentration dropped under 10% in a minute by photocatalysis, and the sorption on both sorbents was even faster. During 20 s, TCS was totally eliminated from the aqueous solution.

Definitely, sorption is not only an environmentally friendly process for contaminant removal, but is also highly efficient. It is obvious that, regarding DCF and TCS, photocatalysis and sorption can be used to remove both pollutants. Finally, the limiting factor always relates to the economy of the process, which is the reason why photocatalysis is hardly applicable on an industrial scale. Therefore, high efficiency, together with the low cost, makes sorption the preferable process.

## 4. Conclusions

Both observed pollutants, TCS and DCF, are confirmed to be present in the water environment, which poses a great risk to water and sediment biota. Toxicity associated with them must be properly assessed and verified, even in connection with the application of treatment technology to prevent formation of toxic intermediates. Furthermore, it is essential to select an appropriate testing organism due to the fact that particular organisms (used even within the standardized techniques for measuring acute toxicity) may show different results. In the case of our research, DCF was agreed on and the conclusions related to it confirmed the results of previous studies. However, in the case of TCS, the measured toxicity was lower. Moreover, it appeared that the use of alginate beads for toxicity testing could not be fully supported. Concerning living free alga and immobilized alga *R. subcapitata*, comparable results were achieved only for DCF, whereas no agreement was reached related to TCS and the reference substance (K_2_Cr_2_O_7_).

The observed treatment processes (i.e., photolysis, photocatalysis and sorption) confirmed their high and fast efficiency for both aimed pollutants. However, in the case of photolysis, toxic intermediates were observed to be formed (toxicity assessed by the *A. fischeri* bioluminescence test), which, consequently, led to this process exclusion. Additionally, the sorption of DCF on Norit, as well as the removal by photocatalysis, was much faster than on SuperSorbon. Finally, due to the high costs of the photocatalysis process, sorption is a favorable solution for removing these pharmaceutical and personal care products from water sources. With respect to its low cost, it could also be used for small wastewater treatment plants, which usually cover less than 10% of the population; however, they represent a large area, for example, in the EU, 75–80%.

## Figures and Tables

**Table 1:** Textural properties of sorbents and TiO_2_ photocatalyst.

Tested Materials	Textural Characteristics			
	S_BET_ (m^2^·g^−1^)	S_meso_ (m^2^·g^−1^)	V_micro_ (mm^3^_liq_·g^−1^)	r_max_ (nm)
Norit	688	274	216	4.7
SuperSorbon	1378	612	395	7.6
TiO_2_	68	50	14	1.5

**Table 2:** Results of tests with the living (free) culture of *R. subcapitata*.

Pollutant	Concentration (mg·L^−1^)	Average Growth Rate ∗ (d^−1^)			Average Inhibition Rate ∗ (%)		
Potassium dichromate (K_2_Cr_2_O_7_)	0	1.68	1.84	1.95	-	-	-
	3.0 × 10^−1^	1.71	1.79	1.73	−2	3	11
	6.0 × 10^−1^	1.51	1.48	1.81	10	19	7
	12.0 × 10^−1^	1.09	1.15	1.70	35	37	12
	24.0 × 10^−1^	0.59	0.54	0.61	65	71	69
	48.0 × 10^−1^	0.53	0.52	0.42	68	72	78
Triclosan (TCS)	0	1.71	1.84	1.71	-	-	-
	1.5 × 10^−3^	1.85	1.79	1.84	−8	3	−8
	4.5 × 10^−3^	0.73	1.18	0.72	58	36	58
	13.5 × 10^−3^	0.53	0.98	0.52	69	46	70
	40.5 × 10^−3^	0.37	0.91	0.39	78	50	77
	121.5 × 10^−3^	0.46	0.58	0.47	73	69	73
Diclofenac (DCF)	0	1.67	1.85	1.95	-	-	-
	0.5 × 10^2^	1.55	1.62	1.63	7	12	16
	1.0 × 10^2^	1.12	0.81	1.59	33	56	18
	2.0 × 10^2^	0.72	0.74	0.92	57	60	52
	4.0 × 10^2^	0.68	0.78	0.57	59	58	71
	8.0 × 10^2^	0.62	0.72	0.58	63	61	70

Note: ∗ three columns mean three replicates.

**Table 3:** Results of tests with *R. subcapitata* in the alginate (immobilized) form.

Pollutant	Concentration (mg·L^−1^)	Average Growth Rate ∗ (d^−1^)			Average Inhibition Rate ∗ (%)		
Potassium dichromate (K_2_Cr_2_O_7_)	0	1.90	1.68	1.84	-	-	-
	3.0 × 10^−1^	1.76	1.80	1.79	8	-8	3
	6.0 × 10^−1^	1.38	1.45	1.47	28	13	20
	12.0 × 10^−1^	0.69	0.69	0.59	64	59	68
	24.0 × 10^−1^	0.34	0.39	0.36	82	77	80
	48.0 × 10^−1^	0.28	0.44	0.25	85	74	86
Triclosan (TCS)	0	1.90	1.68	1.84	-	-	-
	1.5 × 10^−3^	1.53	1.74	1.71	20	−4	7
	4.5 × 10^−3^	0.78	1.12	0.92	59	33	50
	13.5 × 10^−3^	0.53	0.57	0.55	72	66	70
	40.5 × 10^−3^	0.38	0.39	0.53	80	77	71
	121.5 × 10^−3^	0.36	0.18	0.55	81	89	70
Diclofenac (DCF)	0	1.78	1.67	1.67	-	-	-
	0.5 × 10^2^	1.57	1.50	1.76	12	10	−5
	1.0 × 10^2^	1.37	1.39	1.33	23	16	20
	2.0 × 10^2^	0.80	0.81	0.73	55	51	56
	4.0 × 10^2^	0.68	0.74	0.77	62	55	54
	8.0 × 10^2^	0.61	0.65	0.66	66	61	61

Note: ∗ three columns mean three replicates.

**Table 4:** IC_50_ measured on *R. subcapitata* (free vs. immobilized form).

Pollutant	Living (Free) Algae			Alginate (Immobilized)		
	IC_50_ (mg·L^−1^) ∗ (met1)	IC_50_ (mg·L^−1^) ∗ (met2)	Average IC_50_ (mg·L^−1^)	IC_50_ (mg·L^−1^) ∗ (met1)	IC_50_ (mg·L^−1^) ∗ (met2)	Average IC_50_ (mg·L^−1^)
Potassium dichromate (K_2_Cr_2_O_7_)	1.5913	1.5538	1.6757	0.9302	0.9436	0.9654
	1.4756	1.4734		1.0596	1.0306	
	2.0438	1.9165		0.9355	0.8928	
Triclosan (TCS)	0.00399	0.00359	0.0172	0.00353	0.00344	0.0054
	0.02488	0.02300		0.00815	0.00838	
	0.02483	0.02295		0.00451	0.00456	
Diclofenac (DCF)	171.60	175.30	177.68	180.64	183.89	189.11
	155.40	155.10		203.20	214.20	
	203.45	205.25		168.70	184.00	

Note: ∗ three rows mean three replicates.

## Data Availability

Not applicable.
